# Homologous and Heterologous Prime-Boost Vaccination: Impact on Clinical Severity of SARS-CoV-2 Omicron Infection among Hospitalized COVID-19 Patients in Belgium

**DOI:** 10.3390/vaccines11020378

**Published:** 2023-02-07

**Authors:** Marjan Meurisse, Lucy Catteau, Joris A. F. van Loenhout, Toon Braeye, Laurane De Mot, Ben Serrien, Koen Blot, Emilie Cauët, Herman Van Oyen, Lize Cuypers, Belgian Collaborative Group on COVID-19 Hospital Surveillance, COVID-19 Genomics Belgium Consortium, Annie Robert, Nina Van Goethem

**Affiliations:** 1 Department of Epidemiology and public health, Sciensano, 1070 Brussels, Belgium; 2 Department of Epidemiology and Biostatistics, Institut de Recherche Expérimentale et Clinique, Faculty of Public Health, Université Catholique de Louvain, 1200 Woluwe-Saint-Lambert, Belgium; 3 Department of Public Health and Primary Care, Ghent University, 9000 Ghent, Belgium; 4 Department of Laboratory Medicine, National Reference Center for Respiratory Pathogens, University Hospitals Leuven, 3000 Leuven, Belgium; 5 Laboratory of Clinical Microbiology, Department of Microbiology, Immunology and Transplantation, KU Leuven, 3000 Leuven, Belgium

**Keywords:** COVID-19 vaccination, clinical severity, SARS-CoV-2 variants

## Abstract

We investigated effectiveness of (1) mRNA booster vaccination versus primary vaccination only and (2) heterologous (viral vector–mRNA) versus homologous (mRNA–mRNA) prime-boost vaccination against severe outcomes of BA.1, BA.2, BA.4 or BA.5 Omicron infection (confirmed by whole genome sequencing) among hospitalized COVID-19 patients using observational data from national COVID-19 registries. In addition, it was investigated whether the difference between the heterologous and homologous prime-boost vaccination was homogenous across Omicron sub-lineages. Regression standardization (parametric g-formula) was used to estimate counterfactual risks for severe COVID-19 (combination of severity indicators), intensive care unit (ICU) admission, and in-hospital mortality under exposure to different vaccination schedules. The estimated risk for severe COVID-19 and in-hospital mortality was significantly lower with an mRNA booster vaccination as compared to only a primary vaccination schedule (RR = 0.59 [0.33; 0.85] and RR = 0.47 [0.15; 0.79], respectively). No significance difference was observed in the estimated risk for severe COVID-19, ICU admission and in-hospital mortality with a heterologous compared to a homologous prime-boost vaccination schedule, and this difference was not significantly modified by the Omicron sub-lineage. Our results support evidence that mRNA booster vaccination reduced the risk of severe COVID-19 disease during the Omicron-predominant period.

## 1. Introduction

Coronavirus disease 2019 (COVID-19), caused by the severe acute respiratory syndrome coronavirus 2 (SARS-CoV-2), has led to a public health crisis across the world. The clinical severity of COVID-19 ranges from asymptomatic, mild or moderate (e.g., experiencing fever, headache, diarrhea, and myalgia) to severe disease (e.g., experiencing dyspnea, hypoxemia, acute respiratory distress syndrome (ARDS)) [1]. SARS-CoV-2 vaccination has shown to provide effective protection against severe SARS-CoV-2 infections requiring admission to the hospital [2,3,4,5,6] or intensive care unit (ICU) [6,7], and COVID-19-related death [2,3,5]. In Belgium, a large-scale vaccination campaign was initiated on 5 January 2021 [8,9] followed by the roll out of a booster vaccination campaign from September 2021 onwards, resulting in 79% of the total population with a primary vaccination schedule, 62% with a booster dose, and 33% with two booster doses by the beginning of December 2022 [10]. The BNT162b2 vaccine (Comirnaty^®^, Pfizer–BioNTech), mRNA-1273 vaccine (Spikevax^®^, Moderna), Ad26.COV2.S vaccine (COVID-19 vaccine Janssen^®^, Johnson & Johnson) and ChAdOx1-S vaccine (Vaxzevria^®^, Oxford–AstraZeneca) were initially approved by the European Medicines Agency (EMA), and have widely been administered for primary vaccination throughout the Belgian population [10]. Different types of vaccines exist, each training the human immune system in a different way [11]. Pfizer an Moderna vaccines are mRNA vaccines (i.e., artificially created viral mRNA), while the latter two are viral vector vaccines (i.e., genetic material of the virus placed in a viral vector) [12,13,14,15]. MRNA vaccines BNT162b2 and mRNA-1273 were primarily used for booster vaccination in Belgium [10], potentially matching the vaccine type (mRNA or viral vector) of the primary vaccination (i.e., a homologous booster) or not (i.e., a heterologous booster). SARS-CoV-2 vaccines act through humoral immune responses (e.g., B-cell proliferation into neutralizing antibody-producing plasma cells, neutralizing antibodies target the viral spike protein and block the virus’ ability to enter human cells), cellular immune responses (e.g., T cell proliferation into cytotoxic T cells) and memory responses of B- and T-cells [11,16]. It has been previously indicated for non-SARS-CoV-2 vaccines that heterologous prime-boost vaccination might result in more durable and broader vaccine-induced immune response and a higher number of neutralizing antibodies [17,18]. The same has been suggested for SARS-CoV-2 vaccines [19,20,21]; however, this might differ according to circulating SARS-CoV-2 variants, and the vaccine types and order of vaccine types used for primary and booster vaccination [17,20]. To our knowledge, the literature comparing the effectiveness of SARS-CoV-2 ‘viral vector–mRNA’ and ‘mRNA–mRNA’ prime-boost vaccination during a period of Omicron circulation is still limited.

At the same time, emerging SARS-CoV-2 variants accumulate mutations over time, potentially resulting in viral characteristics that allow them to more efficiently evade vaccine-induced immunity. A mismatch between the variant and the vaccine may subsequently reduce the efficacy of the vaccine [22,23]. For example, due to its large number of mutations in the viral spike gene (i.e., the target of neutralizing antibodies), the Omicron variant (lineage B.1.1.529) was found to be more efficient in evading neutralizing antibodies than previously circulating variants [24,25,26,27] and initial evidence shows a lower effectiveness of booster vaccination against severe clinical outcomes of a Omicron infection compared to previous variants. Hence, after its emergence in late 2021, it has caused significant concerns about the enduring effectiveness of vaccines. Moreover, between sub-lineages of the Omicron variant, characterized by important differences in the S protein of the virus, differences in resistance to neutralization and vaccine effectiveness can be observed [28,29,30]. In Belgium, the initially circulating Omicron BA.1 lineage was first detected in November 2021 [31] and the BA.1 and BA.1.1 lineages jointly represented more than 80% of the positive samples by the first week of January 2022 [32]. Subsequently, BA.2 emerged and gradually replaced BA.1 during early 2022, reaching 80% of circulating strains by the beginning of March 2022 [32]. Next, BA.4 and BA.5 lineages emerged and BA.5 became more prevalent from May 2022 onwards [10,32]. By the beginning of December 2022, BA.5 and its sub-lineages (e.g., BQ.1*) were the predominantly circulating strains [33]. In addition to a potential change in the capacity to evade neutralizing antibodies, the regularly occurring mutations may also alter the viral pathogenicity [34,35]. Therefore, continuously evaluating the impact of newly emerging variants is essential.

The current study aimed to compare the effectiveness of (1) mRNA booster vaccination versus primary vaccination only and (2) a heterologous (viral vector–mRNA) versus homologous (mRNA–mRNA) prime-boost vaccination scheme in preventing severe clinical outcomes of SARS-CoV-2 Omicron infection among hospitalized patients in Belgium, and whether the effect of heterologous versus homologous prime-boost vaccination scheme was homogenous across different Omicron sub-lineages (i.e., is the difference in risk for severe clinical outcomes between heterologous and homologous prime-boost vaccination modified by the Omicron sub-lineage of infection). This study focused on hospitalized COVID-19 patients in Belgium and aims to assess this effect using observational data. By conducting this study, we aim to inform vaccine policy and contribute to knowledge used for the development of strategies for booster campaigns during the continuously evolving COVID-19 epidemic.

## 2. Materials and Methods

### 2.1. Target Trial Specification

This observational study was developed with the aim of emulating two conceptual target randomized control trials comparing the effectiveness of (1) a primary vaccination (viral vector or mRNA) plus mRNA booster vaccination (i.e., homologous or heterologous prime-boost vaccination scheme) versus a primary vaccination (viral vector or mRNA) without booster vaccination and (2) a heterologous (viral vector prime–mRNA boost) versus homologous (mRNA prime–mRNA boost) prime-boost vaccination scheme in preventing severe clinical outcomes of SARS-CoV-2 Omicron infection among hospitalized COVID-19 patients in Belgium. Key elements of the protocol of the two target trials have been specified in Table S1 (Supplementary A).

**Figure 1:**
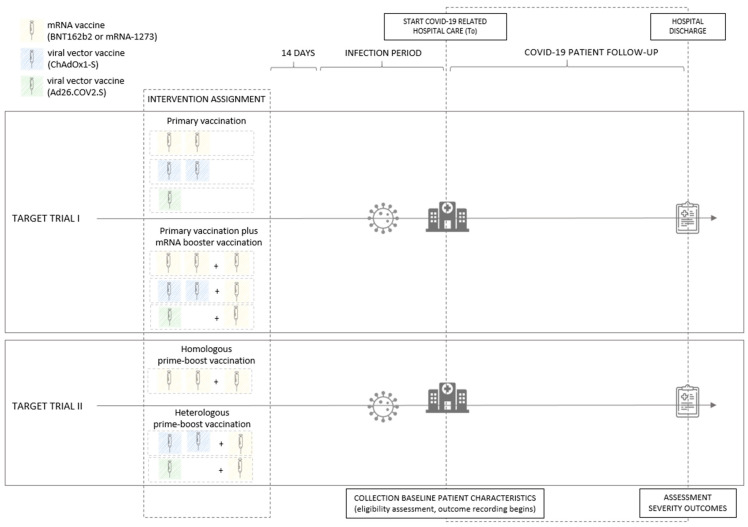
Graphical representation of the conceptual target randomized control trials comparing the effectiveness of (1) primary vaccination plus mRNA booster vaccination versus primary vaccination without booster vaccination (target trial I) and (2) heterologous versus homologous prime-boost vaccination (target trial II), in preventing severe clinical outcomes of SARS-CoV-2 Omicron infection among hospitalized COVID-19 patients in Belgium. Individuals assigned to one of the intervention arms, with a SARS-CoV-2 infection (community- or hospital-acquired, confirmed by an RT-PCR or AG test) acquired at least 14 days after the last administered vaccination dose, requiring COVID-19 related hospital care and fitting with the eligibility criteria, are followed up from the beginning of COVID-19 related hospital care until hospital discharge. Baseline patient characteristics are collected at the start of the COVID-19 related hospital care (T0) and clinical severity (severe COVID-19, ICU admission, and in-hospital mortality) is assessed at hospital discharge.

In both hypothetical target trials, adults (age ≥ 18, with age defined at the start of COVID-19 related hospital care) were randomly assigned to one of the intervention arms. In the first target trial, the intervention arms consist of (a) an mRNA (two doses of BNT162b2 or mRNA-1273) or viral vector (one dose of Ad26.COV2.S or two doses of ChAdOx1-S) primary vaccination plus an mRNA (BNT162b2 or mRNA-1273) booster vaccination and (b) an mRNA or viral vector primary vaccination without booster vaccination (see Figure 1). In the second target trial the intervention arms consist of (a) heterologous prime-boost vaccination, defined as a viral vector (one dose of Ad26.COV2.S or two doses of ChAdOx1-S) primary vaccination plus mRNA (BNT162b2 or mRNA-1273) booster vaccination, and (b) homologous prime-boost vaccination, defined as an mRNA (two doses of BNT162b2 or mRNA-1273) primary vaccination plus mRNA (BNT162b2 or mRNA-1273) booster vaccination (see Figure 1). Individuals assigned to one of the intervention arms were only eligible for inclusion in the study if they subsequently acquire a SARS-CoV-2 infection (either community- or hospital-acquired) that is confirmed by a reverse transcription polymerase chain reaction (RT-PCR) or antigen (AG) laboratory test and, in addition, if the COVID-19 disease progression of this infection requires COVID-19 related hospital care. Individuals are not eligible when the detected SARS-CoV-2 infection occurs less than 14 days after the last vaccination dose of their vaccination schedule and when the lineage assigned based on a whole genome sequencing (WGS) test result indicated infection with a variant other than Omicron. Patient follow up for eligible patients starts at the beginning of COVID-19 related hospital care (time zero, T_0_) and ends at hospital discharge. Baseline patient characteristics were collected at the start of the COVID-19 related hospital care, and clinical severity (severe COVID-19, ICU admission, and in-hospital mortality) was assessed at hospital discharge.

### 2.2. Target Trial Emulation

Observational data from existing national surveillance systems and administrative sources were leveraged to emulate the specified hypothetical target trials.

#### 2.2.1. Data Sources

Secure individual-level linkage of selected variables from different existing data sources was conducted within the framework of the LINK-VACC project, initiated by Sciensano, the Belgian national institute for health. Linkage was executed using a pseudonymized national registry number within a secured environment (HealthData.be platform). Data on Belgian hospitalized COVID-19 patients registered in the Clinical Hospital Survey (CHS, including information on comorbidities and severity indicators) [36] were linked to data from the COVID-19 TestResult database [37,38,39] (to obtain information on reinfections and the variant of infection), data from the national vaccine registry (Vaccinnet+) [40], data on socio-economic factors obtained from the Belgian Statistical Office (StatBel), data from the surge capacity survey (to obtain information on the hospital bed occupancy) [36] and data from the Common Base Registry for Healthcare Actor (CoBRHA, to obtain information on the recognition and activity of people as healthcare actors) [41]. The data infrastructure has been described elsewhere [42].

#### 2.2.2. Eligibility Criteria

The study population consisted of symptomatic laboratory-confirmed COVID-19 patients admitted to a Belgian hospital between 1 September 2021 (i.e., the start of the Belgian booster vaccination campaign) and 23 November 2022 (i.e., the date of data extraction minus two weeks to allow for sufficient follow-up time), with an admission form reported in the CHS. To comply with the eligibility criteria of the target trial, hospitalized patients who were tested for COVID-19 in a screening context were excluded, as well as patients diagnosed with COVID-19 more than 20 days before hospital admission or after hospital discharge, with the aim of only considering patients who received hospital care for their COVID-19 related pathology and excluding patients who had reported severe clinical outcomes unrelated to COVID-19. Furthermore, underaged patients (<18 years) were excluded, as well as transferred or readmitted patients, and patients admitted to a psychiatric hospital or a hospital without an ICU. Patients with a missing pseudonymized national registry number were additionally excluded, as this information was required for the linkage to other registries. Furthermore, patients with an unknown date of diagnosis, starting date of COVID-19-related hospital care or date of discharge were not included. As we assessed the risk of severe clinical outcomes of a SARS-CoV-2 Omicron infection under exposure to different vaccination schedules, only patients infected with Omicron were considered for the analysis. Four sub-lineages of Omicron could be identified in the eligible patients by linking WGS-confirmed lineage data from COVID-19 TestResult database: BA.1, BA.2, BA.4 and BA.5. Due to the low prevalence of BA.4 infections among the eligible patients with an WGS-confirmed Omicron infection (13 out of 1119 patients) and co-circulation of BA.4 and BA.5 in time, BA.4 and BA.5 were combined and further referred to as BA.4/5. For the main analysis, infection with an Omicron variant was ascertained through the linkage with the WGS-confirmed lineage data from the COVID-19 TestResult database. Patients without a linked WGS-confirmed lineage or with a linked WGS-confirmed lineage different from Omicron BA.1, BA.2, BA.4 or BA.5 were excluded from the study population in the main analysis. As linkage with the non-exhaustive WGS test results limited the sample size significantly (only for 11% of the eligible patients a WGS-confirmed lineage could be linked) and might have resulted in selection bias [43], a sensitivity analysis was performed where patients were categorized based on their COVID-19 diagnosis date within time periods with known circulation of the Omicron sub-lineages [33]. Patients diagnosed with COVID-19 during a restricted time period with an approximated circulation of BA.1, BA.2 or BA.4/5 of more than 80%, were assigned to the BA.1, BA.2 or BA.4/5 Omicron sub-lineage, respectively. These restricted time periods were set by considering the distribution of variants in the representative Belgian baseline genomic surveillance [33]. The time periods to assign the BA.1, BA.2 or BA.4/5 Omicron sub-lineage were set to 3 January 2022–13 February 2022, 14 March 2022–22 May 2022 and later than 22 June 2022, respectively. Patients with a COVID-19 diagnosis date outside these predefined periods were excluded from the study population in the sensitivity analysis.

#### 2.2.3. Intervention Strategies and Assignment

In the population eligible for inclusion, intervention groups could be determined through individual-level linkage with data from the national vaccine registry (Vaccinnet+). Vaccination status of eligible patients was assigned on the date of the COVID-19 diagnosis resulting in the hospitalization.

To emulate the target trials, patients who had completed one of the vaccination schedules as defined in the intervention arms and who had received the last dose of their vaccination schedule at least 14 days before the date of diagnosis of the confirmed COVID-19 infection were assigned to the respective intervention group. Patients with a vaccination schedule not corresponding to the definition of one of the intervention arms, were excluded from the study population (e.g., not or partially vaccinated patients, patients who received a heterologous primary vaccination scheme, patients who had received more than one booster dose, patients who received a non-mRNA booster vaccination).

**Figure 2:**
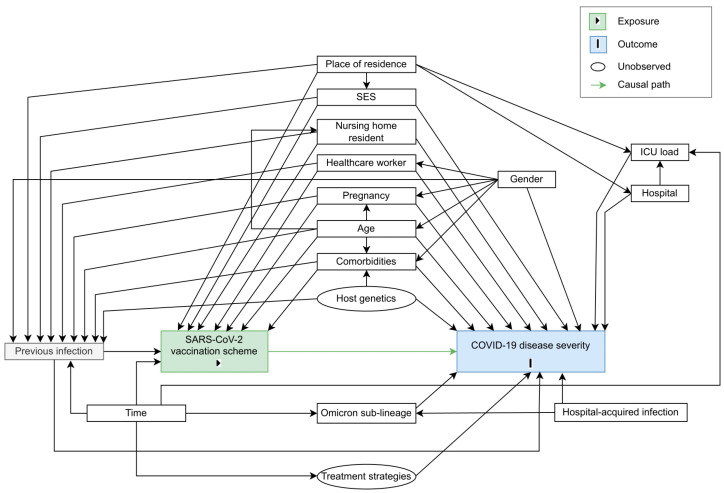
Directed acyclic graph (DAG) representing the causal assumptions that were made when assessing the effect of SARS-CoV-2 vaccination scheme (i.e., the exposure, indicated in green) on COVID-19 disease severity (i.e., the outcome, indicated in blue). Causal paths are shown as green arrows.

Randomization was mimicked by conditioning on a set of factors that were assumed to confound the exposure-outcome pathway. We assumed that individuals were exchangeable within levels of the confounders. A directed acyclic graph (DAG) was constructed based on a priori assumptions about the causal relationship between COVID-19 vaccination scheme (i.e., the exposure) and COVID-19 disease severity (see Figure 2). The DAG includes both observed and unobserved variables. The code to generate the DAG with the ‘DAGitty’ software [44] is provided in the Supplementary B. A sufficient adjustment set to close the non-causal backdoor paths captured in the DAG was identified: patients’ comorbidities, age, gender, pregnancy status, host genetics, socio-economic status (SES), previous infection(s), whether the patient is a nursing home resident, whether the patient is a healthcare worker, the hospital of admission and ICU load during COVID-19-related hospital stay, whether the patient had a hospital-acquired infection, treatment strategies used during the time of admission, and Omicron sub-lineage of infection.

The sufficient adjustment set, determining which covariates we should ideally consider to close the non-causal backdoor paths as captured in the DAG, was approximated by information available in the different registries within the LINK-VACC project.

Individual comorbidities, registered in the CHS, were grouped into three categories based on their strength as a risk factor for severe COVID-19: no underlying comorbidities, medium-risk underlying comorbidities (i.e., cardiovascular disease, chronic liver disease, obesity, immunosuppressive therapy, cognitive disorder, diabetes, or chronic lung disease) and high-risk comorbidities (i.e., solid cancer, hematological cancer, chronic kidney disease, immunodeficiency, chronic neurological disease, or people with an organ transplant). SES was approximated by education level and income categories at the individual level, and by the median net taxable income per capita and population density of the postal code of residence. Education level categories were obtained by categorizing the assigned International Standard Classification of Education (ISCED) levels into ‘low’ (ISCED0–ISCED2), ‘medium’ (ISCED3–ISCED4) and ‘high’ (ISCED5–ISCED8). Income categories were determined by breaking up the net income of the patient’s household into deciles and categorizing these deciles into ‘low’ (decile 1–4), ‘medium’ (decile 5–7) and ‘high’ (decile 8–10). As not only vaccination- but also infection-induced immunity contributes to a patient’s preexisting immunity, previous infection was considered as an important confounder in the analysis. We defined that a patient had a documented previous infection when the sample, for diagnosis of the SARS-CoV-2 infection for which the patient is hospitalized, was collected more than 60 days after the date of sample collection from a previous documented positive COVID-19 test (RT-PCR or AG) [45]. Whether or not a patient was a nursing home resident and whether the infection was nosocomial (i.e., acquired in the hospital) was captured in the CHS. Information on a patient’s activity as a healthcare worker was obtained by combining information from the CoBRHA registry, which contains records on healthcare professionals by degree, and the patient’s admission form in the CHS. The hospital of admission was categorized into three hospital types (general hospitals, university hospitals or general hospitals with a university character). We assumed that treatment strategies were constant over the study period. Information on host genetics was missing.

#### 2.2.4. Follow-up Period and Outcomes of Interest

As in the target trial specification, the outcomes of the causal inference study were (1) the development of severe COVID-19, as defined by the occurrence of either ICU admission, ARDS, or in-hospital mortality (all causes), (2) ICU admission and (3) in-hospital mortality, separately. Patients were followed up from hospital admission until hospital discharge, with details on their COVID-19 disease outcome reported in the discharge form.

### 2.3. Statistical Analyses

Characteristics of patients in the selected study population were presented by their vaccination scheme: primary vaccinated patients (viral vector or mRNA) without booster vaccination, patients who have received a homologous (mRNA–mRNA) prime-boost vaccination scheme, and patients who have received a heterologous (viral vector–mRNA) prime-boost vaccination scheme. Continuous variables were presented by their median value and interquartile range (IQR), while categorical variables were described as a counts (n) and proportions (%).

Missing values in variables included in the multivariable model (see further) were imputed with the R package ‘MICE’ (multivariate imputation via chained equations) [46]. We assumed that the missing values were missing at random (MAR), i.e., missingness can be explained by other covariates and is not related to the missing value itself. Twenty-fold multiple imputation was executed, with predictive mean matching, logistic regression and polytomous logistic regression for numerical, binary and categorical variables, respectively. The variables for severe COVID-19 and nosocomial infection were passively imputed. Twenty iterations were performed to obtain convergence of the Markov chain Monte Carlo (MCMC) algorithm. 

A logistic regression model was built, adjusted for the following covariates: comorbidity group, age, gender, nursing home residence, median net taxable income per capita and population density of the postal code of residence, education level category, income category, previous infection, type of hospital and mean ICU occupancy rate in the hospital of admission during the stay, whether it was a hospital-acquired infection, and a two-way interaction between the Omicron sub-lineage of infection and the vaccination schedule. Covariates for pregnancy status and whether the patient is a healthcare worker were not included in the model because of their low prevalence in the study population, resulting in separation in the regression models. Numeric variables were added both as linear and quadratic term in the model.

Standardization [47] using the fitted logistic regression model (parametric G-formula) was performed in each imputed dataset, where we predict counterfactual risks of potential outcomes under intervention (i.e., under primary vaccination without booster vaccination (R_0_) and primary vaccination plus mRNA booster vaccination (R_1_) in the first emulated target trial, and under homologous (R_0_) and heterologous (R_1_) prime-boost vaccination in the second emulated target trial) using the fitted adjusted logistic regression model. Marginal effect estimates (risk difference, RD = R_1_-R_0_, and relative risk, RR = R_1_/R_0_) were obtained by contrasting the estimated counterfactual risks under each intervention arm. Bootstrapping (with 1000 bootstrap replicates) was performed, using the R package ‘boot’ [48], to obtain the standard error of effect estimates. Rubin’s rules were used to pool the effect estimates and corresponding standard errors from each imputed dataset, to calculate 95% Confidence Intervals (CIs).

Furthermore, we assessed whether the estimated effect of a homologous versus heterologous prime-booster scheme on clinical severity is modified by the Omicron sub-lineage of infection. By fitting a model with the two-way interaction between the Omicron sub-lineage of infection and the vaccination schedule, counterfactual risks of the binary outcomes under exposure of the control or intervention vaccination schedule (primary exposure) and a specific Omicron sub-lineage of infection (secondary exposure) could be estimated (e.g., R_1,BA.1_ indicating the counterfactual risk for a severity outcome under the intervention vaccination schedule—primary vaccination plus mRNA booster vaccination in target trial I and heterologous prime-boost vaccination in target trial II—and when being exposed to the BA.1 lineage, R_0,BA.1_ indicating the counterfactual risk for a severity outcome under the control vaccination schedule—primary vaccination only in target trial I and homologous prime-boost vaccination in target trial II—and when being exposed to the BA.1 lineage). Counterfactual risks under both intervention arms were contrasted under exposure to BA.1 (RD_BA.1_ = R_1,BA.1_-R_0,BA.1_; RR_BA.1_ = R_1,BA.1_/R_0,BA.1_), BA.2 (RD_BA.2_ = R_1,BA.2_-R_0,BA.2_; RR_BA.2_ = R_1,BA.2_/R_0,BA.2_) and BA.4/5 (RD_BA.4/5_ = R_1,BA.4/5_-R_0,BA.4/5_; RR_BA.4/5_ = R_1,BA.4/5_/R_0,BA.4/5_) lineages of infection (for an explanation on the counterfactual definition of effect modification, see the manuscript of Bours [49]). According to the counterfactual definition of effect modification, effect modification can be observed on the additive scale if at least two of the estimated risk differences (RD_BA.1,_ RD_BA.2_ and RD_BA.4/5_) are different and on the multiplicative scale if at least two of the estimated relative risks (RR_BA.1_, RR_BA.2_, RR_BA.4/5_) are different.

## 3. Results

### 3.1. Characteristics of the Study Population

**Figure 3:**
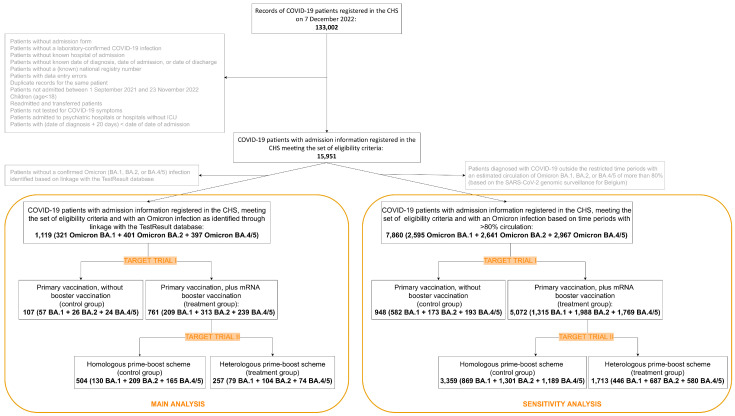
Flow chart representing the study selection procedures.

On 7 December 2022 (i.e., the date of data extraction), 133,002 admission forms were registered in the CHS. From these, 117,051 records of patients not meeting the eligibility criteria or duplicate records of the same patient were excluded. This resulted in 15,951 unique patient records with available admission information and meeting the eligibility criteria. The study population selection procedures for the main and sensitivity analysis of both emulated trials are graphically represented in Figure 3.

#### 3.1.1. Main Analysis

For 1119 out of the 15,951 patients, the Omicron variant of infection was assigned based on linkage to WGS-confirmed lineage data. From those, 868 had a vaccination schedule corresponding to one of the intervention arms of target trial I or II and were subsequently included in the analysis. They were admitted to the hospital for COVID-19 between 16 December 2021 and 10 November 2022. In the study population, 59% (513/868) were male, 11% (99/868) were a nursing home resident, and median age was 79 years (IQR: 70–86). 3% (29/868) of the patients in the study population had a documented previous infection and 56.9% (494/868) had at least one underlying comorbidity characterized by a high risk for severe COVID-19.

Patient characteristics by vaccination scheme (primary vaccinated patients versus patients with a homologous prime-boost vaccination scheme versus patients with a heterologous prime-boost vaccination scheme) are presented in Table 1. Patients who had received a booster vaccine dose in our study population tended to be older, more frequently were male and nursing home resident and more frequently had certain comorbidities (e.g., cardiovascular disease, arterial hypertension, chronic renal disease), less frequently had an identified previous infection, and more frequently were characterized by severe clinical COVID-19 outcomes (e.g., ICU admission, in-hospital mortality), compared to patients with only a primary vaccination schedule. Patient characteristics between patients who received a homologous and heterologous prime-boost vaccination scheme were more similar.

#### 3.1.2. Sensitivity Analysis

For 7860 out of the 15,951 patients, the Omicron sub-lineage of infection was assigned based on their date of COVID-19 diagnosis, falling within pre-defined time periods with known circulation of the Omicron BA.1, BA.2 and BA.4/5 sub-lineages. From those, 6020 had a vaccination schedule corresponding to one of the intervention arms of target trial I or II and were subsequently included in the analysis. They were admitted to the hospital for COVID-19 between 18 October 2021 and 23 November 2022. In the study population, 52% (3133/6020) were male, 14% (814/6020) were a nursing home resident, and median age was 78 years (IQR: 67–86). 6.2% (373/6020) of the patients in the study population had a documented previous infection and 47.4% (2852/6020) had at least one underlying comorbidity characterized by a high risk for severe COVID-19.

### 3.2. Effect Estimates

#### 3.2.1. Target Trial I: Primary Vaccination Plus mRNA Booster Vaccination versus Primary Vaccination without Booster Vaccination

##### Main Analysis

The effect estimates (RDs and RRs) for the three considered clinical outcomes (severe COVID-19, ICU admission, in-hospital mortality) in the study population, are presented in Table 2. The estimated counterfactual risk for severe COVID-19 (defined based on a combination of severity indicators, including ARDS, ICU admission, and in-hospital mortality) among hospitalized COVID-19 patients in Belgium infected with the BA.1, BA.2, or BA.4/5 Omicron sub-lineage (as identified through linkage with WGS-confirmed lineage data) was significantly lower when having received an mRNA booster vaccination compared to when only having received a primary vaccination schedule (RD = −0.13 [−0.24; −0.01], RR = 0.59 [0.33; 0.85]). In addition, when only looking at in-hospital mortality as severity indicator, the estimated risk was significantly lower when having received the booster dose (RD = −0.13 [−0.25; 0.00], RR = 0.47 [0.15; 0.79]). There was no significant difference in counterfactual risk for ICU admission (RD = −0.04 [−0.11; 0.04], RR = 0.74 [0.22; 1.26]).

##### Sensitivity Analysis

The effect estimates (RDs and RRs) for the three considered clinical outcomes (severe COVID-19, ICU admission, in-hospital mortality) in the study population are presented in Table S2 (Supplementary C). As in the main analysis, in the sensitivity analysis, the estimated counterfactual risk for severe COVID-19 (defined based on a combination of severity indicators, including ARDS, ICU admission, and in-hospital mortality) and in-hospital mortality among hospitalized COVID-19 patients in Belgium infected with the BA.1, BA.2, or BA.4/5 Omicron sub-lineage (as defined by the time period of COVID-19 diagnosis) was significantly lower when having received an mRNA booster vaccination as compared to when only having received a primary vaccination schedule (severe COVID-19: RD = −0.05 [−0.08; −0.02], RR = 0.75 [0.61; 0.88]; in-hospital mortality: RD = −0.05 [−0.08; −0.02], RR = 0.67 [0.51; 0.82]). No significant difference in standardized risk for ICU admission was observed (RD = −0.01 [−0.03; 0.01], RR = 0.87 [0.63; 1.11]).

#### 3.2.2. Target Trial II: Heterologous versus Homologous Prime-Boost Vaccination

##### Main Analysis

The effect estimates (RDs and RRs) for the three considered clinical outcomes (severe COVID-19, ICU admission, in-hospital mortality), overall and under exposure to Omicron sub-lineage BA.1, BA.2 and BA.4/5, separately, are presented in Table 3. No significance difference was observed in estimated counterfactual risk for severe COVID-19 (defined based on a combination of severity indicators, including ARDS, ICU admission, and in-hospital mortality), ICU admission and in-hospital mortality among hospitalized COVID-19 patients in Belgium infected with the BA.1, BA.2 or BA.4/5 Omicron sub-lineage (as identified through linkage with WGS-confirmed lineage data) when having received a heterologous prime-boost vaccination as compared to when having received a homologous schedule (severe COVID-19: RD = −0.04 [−0.10; 0.02], RR = 0.80 [0.53; 1.07]; ICU admission: RD = −0.03 [−0.07; 0.02], RR = 0.73 [0.34; 1.12]; in-hospital mortality: RD = 0.00 [−0.05; 0.04], RR = 0.96 [0.55; 1.38]).

Furthermore, we assessed whether this estimated effect was homogenous across different Omicron sub-lineages. We observed no significant additive and multiplicative modification by the Omicron sub-lineage of the effect of the vaccination schedule (heterologous versus homologous prime-boost vaccination) on severe COVID-19, ICU admission, and in-hospital mortality, since none of the estimated risk differences and relative risks were significantly different from each other (see Table S3, Supplementary D).

##### Sensitivity Analysis

The effect estimates (RDs and RRs) for the three considered clinical outcomes (severe COVID-19, ICU admission, in-hospital mortality), overall and under exposure to Omicron sub-lineage BA.1, BA.2 and BA.4/5 separately, are presented in S4 (Supplementary E). No significance difference was observed in estimated counterfactual risk for severe COVID-19 (defined based on a combination of severity indicators, including ARDS, ICU admission, and in-hospital mortality), ICU admission and in-hospital mortality among hospitalized COVID-19 patients in Belgium infected with the BA.1, BA.2 or BA.4/5 Omicron sub-lineages (as defined by the time period of COVID-19 diagnosis) when having received a heterologous prime-boost vaccination as compared to having received a homologous schedule (severe COVID-19: RD = 0.01 [−0.01; 0.03], RR = 1.06 [0.91; 1.21]; ICU admission: RD = 0.00 [−0.01; 0.02], RR = 1.02 [0.78; 1.26]; in-hospital mortality: RD = 0.00 [−0.02; 0.02], RR = 1.01 [0.83; 1.19]). Furthermore, we observed no significant additive and multiplicative modification by the Omicron sub-lineage of the effect of the vaccination schedule (heterologous versus homologous prime-boost vaccination) on severe COVID-19, ICU admission and in-hospital mortality, since none of the estimated risk differences and relative risks were significantly different from each other (see Table S5, Supplementary F).

## 4. Discussion

In this study, we compared the effectiveness of (1) an mRNA booster vaccine versus primary vaccination only and (2) a heterologous (viral vector–mRNA) versus homologous (mRNA–mRNA) prime-boost vaccination in preventing severe clinical outcomes of SARS-CoV-2 Omicron infection (BA.1, BA.2 or BA.4/5 Omicron sub-lineage) among hospitalized COVID-19 patients in Belgium, and whether this effect was homogenous across the different Omicron sub-lineages. This study was conducted using observational data from national COVID-19 registries.

We observed that the administration of a booster dose (mRNA vaccine) in addition to a homogenous primary vaccination (any type) schedule significantly reduced the estimated risk of progression of an BA.1, BA.2 or BA.4/5 Omicron infection to severe COVID-19 (experiencing an ARDS event, or ICU transfer, or in-hospital mortality) and in-hospital mortality in the population of patients treated for COVID-19 in a Belgian hospital. This is in line with results from previous studies, in which the effectiveness of an mRNA booster vaccination against severe Omicron infections was found to increase, compared to patients with only a primary vaccination [26,50,51]. Therefore, based on previous and the current results, in a period of predominant Omicron circulation, the administration of a booster vaccine dose can be recommended. We observed a similar, though weaker and non-significant, trend in effectiveness of mRNA booster vaccination against ICU admission of patients treated for COVID-19 in a Belgian hospital. This might be due to a lack of statistical power, resulting from small sample sizes. Furthermore, in contrast to severity outcomes such as mortality or ARDS, ICU admission rather reflects clinical decision making which partly depends on contextual factors (such as therapeutic guidelines in place and varying healthcare capacity). As the median age was 79 and 84 years in the cohort with a homologous and heterologous prime-boost vaccination schedule respectively, (versus 68 years among those that received a primary vaccination schedule only), it could be that this older patient population was less likely to be admitted into ICU due to perceived clinical futility. Further investigations in a younger population (e.g., excluding patients older than 85 years old) could be carried out in the future.

No difference in estimated risk of severe BA.1, BA.2 or BA.4/5 Omicron infection was observed between a homologous and heterologous prime-booster scheme with an mRNA booster vaccine in the study population. Early results suggested that mixing of vaccine types for primary and booster vaccination might result in a stronger immune response [19,20,21] and therefore might offer higher protection against severe COVID-19. However, this was not observed in our study population of hospitalized COVID-19 patients in Belgium and infected with Omicron BA.1, BA.2 or BA.4/5. This might be related to the SARS-CoV-2 lineages we consider in this study, namely Omicron BA.1, BA.2, BA.4 or BA.5, characterized by their ability to more efficiently evade vaccine-induced neutralizing antibodies compared to previously circulating variants [24,25,26,27]. Furthermore, in this study we compare the effectiveness of SARS-CoV-2 ‘viral vector–mRNA’ and ‘mRNA–mRNA’ prime-boost vaccination and results might depend on the vaccine types and order of the vaccine types used for primary and booster vaccination. Previously, there might have been a concern for less protection against severe COVID-19 disease in people vaccinated with the less effective Ad26.COV2.S and ChAdOx1-S vaccines [52]; however, our results indicate that after booster vaccination with the BNT162b2 or mRNA-1273 vaccine, the type of vaccine used for primary vaccination might no longer be of concern and ‘viral vector–mRNA’ and ‘mRNA–mRNA’ prime-boost vaccination schemes might provide similar protection against severe Omicron infection for hospitalized COVID-19 patients in Belgium. We found, during the circulation of BA.1, BA.2 and BA.4/5 Omicron sub-lineages, no evidence of a difference in protection against severe clinical outcomes between homologous and heterologous prime boost between Omicron sub-lineages.

This study has several important strengths. First, detailed patient information was available through the CHS, to which data on socio-economic characteristics of the patients, previous documented infections, sequencing results for identification of the SARS-CoV-2 Omicron sub-lineage of infection, and exhaustive data on administered vaccine doses could be linked. This allowed us to control for many of the identified confounders, making our effect estimates more reliable and limiting confounder bias. Second, even though the CHS surveillance is not exhaustive, hospitals reporting to the CHS are geographically distributed over the country, resulting in an increased generalizability of the results for the entire population of hospitalized COVID-19 patients in Belgium. Third, we take into account different clinical severity outcomes of COVID-19, including a composite measure (i.e., severe COVID-19, defined as experiencing an acute respiratory distress syndrome (ARDS) event, or an ICU admission, or in-hospital mortality), a severity measure dependent on clinical decision making (i.e., ICU admission), and a ‘hard’ objective severity measure (i.e., in-hospital mortality). These different outcomes might capture different aspects of vaccine effectiveness and might inform policy in an alternative way.

There are also some limitations to the study. First, in the main analysis, the variant of infection was assigned based on linkage with WGS-confirmed lineage data from the COVID-19 TestResult database. This might result in selection bias and drastically limits the sample size [43]. The small sample sizes reduce the statistical power and the reliability of significant findings [53]. Furthermore, the lineage data obtained from the COVID-19 TestResult database might be slightly biased due to delayed adjustment of the data entry tool when new sub-lineages were defined. A sensitivity analysis was conducted where the variant of infection was assigned based on the timing of diagnosis during restricted time periods with a predominant circulation of BA.1, BA.2 and BA.4/5. Conclusions from this sensitivity analysis remain the same and are not affected by the method of lineage assignment, indicating the robustness of our findings. Second, the DAG was constructed and a sufficient adjustment set was determined based on a priori assumptions about the causal relationship between the exposure and outcome. Hence, there might exist residual bias from unidentified confounders. Furthermore, host genetics was identified as a confounder in the DAG, but was unobserved, and we were not able to adjust for this variable. In addition, the sufficient adjustment set was approximated by available information. For example, the considered previous infections entail only documented previous infections, which is dependent on the testing strategy and care-seeking behavior. Furthermore, alternative definitions for a reinfection can be adopted [45,54]. Our definition of a reinfection was based on the recommendations of the European Center for Disease Prevention and Control (ECDC) to take a minimal period of 60 days to elapse since the previous documented infection to consider an infection as a reinfection [45]. The hospital to which the patient admitted was identified as a confounder and was taken into account by adjusting for the hospital type, not the individual hospital, since (due to the small sample size) including the individual hospital in the models resulted in complete separation. Treatment strategies were assumed to be constant during the study period; however, if this assumption is not correct, adjustment for this confounder is required. Hence, residual confounding bias might be present in the effect estimates. We also have to account for the possibility of model misspecification, while no model misspecification is an assumption for obtaining unbiased estimates through standardization. Third, to investigate if the estimated effect of a homologous versus heterologous prime-booster scheme on clinical severity is modified by the Omicron sub-lineage of infection, the Omicron BA.4 and BA.5 lineages were combined. Viral characteristics (e.g., virulence and transmissibility) of these variants might differ; however, due to the small sample size and low prevalence of BA.4 infections, we were not able to separate the latter sub-lineage in our analysis. However, the BA.4 and BA.5 lineages were co-circulating in time and the Omicron sub-lineage was not our primary exposure of interest. Fourth, we aimed to only include hospitalized patients registered in the CHS that are treated for COVID-19; however, it is difficult to deduce for the collected data in the CHS for which reason a patient was treated in the hospital. Furthermore, the cause of the documented severe clinical outcomes was difficult to assess from the available data. Fifth, for performing multiple imputation of missing values, the assumption of missingness at random was made. However, the underlying missing data mechanism is unknown. Sixth, different selection steps were performed to obtain data of our study population (i.e., selection of patients tested for COVID-19, treated in the hospital for COVID-19 symptoms, registered in the CHS, and with an Omicron BA.1, BA.2, or BA.4/5 infection identified by linking WGS-confirmed lineage data) [43]. Hence, patients in our study population might have specific characteristics distinctive from the population of all hospitalized patients in Belgium and the general population, thereby limiting generalization. It is important to take into account these study selection procedures when interpreting the estimated counterfactual risks and effect estimates (RDs and RRs) which are tied to the particular population across which the marginalization was carried out.

## 5. Conclusions

Our results support evidence that the administration of an mRNA booster vaccine reduces the risk of severe COVID-19 disease among hospitalized patients in a time with predominant Omicron circulation, and suggest that there is no difference in protection offered by the contrasted prime-boost vaccination schemes. These results can inform vaccine policy in Belgium, and contribute to knowledge used for the development of strategies for booster campaigns during the continuing COVID-19 epidemic.

## Figures and Tables

**Table 1:** Characteristics by vaccination scheme (primary vaccinated patients versus patients with a homologous prime-boost vaccination scheme *versus* patients with a heterologous prime-boost vaccination scheme) within a multi-center study to assess the effect of SARS-CoV-2 vaccination scheme on clinical outcomes among hospitalized COVID-19 patients.

	Primary Vaccination (No Booster) (n = 107)	Primary Vaccination, Plus mRNA Booster Vaccination	
		Homologous Prime-Boost Vaccination (n = 504)	Heterologous Prime-Boost Vaccination (n = 257)
	Number/Total Number (Percent)		
Demographics			
Age (years), median (IQR)	68 (51–80)	79 (72–85)	84 (74–89)
Male gender, n/N (%)	60/107 (56.1)	300/504 (59.5)	153/257 (59.5)
Nursing home resident, n/N (%)	5/106 (4.7)	68/497 (13.7)	26/256 (10.2)
Healthcare worker, n/N (%)	1/105 (1.0)	4/500 (0.8)	3/256 (1.2)
Comorbidities			
Cardiovascular Disease, n/N (%)	36/107 (33.6)	277/504 (55.0)	151/257 (58.8)
History of Arterial Hypertension, n/N (%)	39/107 (36.4)	218/504 (43.3)	104/257 (40.5)
Diabetes mellitus, n/N (%)	27/107 (25.2)	142/504 (28.2)	70/257 (27.2)
Obesity, n/N (%)	8/107 (7.5)	39/504 (7.7)	23/257 (8.9)
Chronic Pulmonary Disease, n/N (%)	28/107 (26.2)	119/504 (23.6)	74/257 (28.8)
Chronic Neurological Disease, n/N (%)	21/107 (19.6)	111/504 (22.0)	40/257 (15.6)
Chronic Cognitive Deficit, n/N (%)	15/107 (14.0)	65/504 (12.9)	28/257 (10.9)
Chronic Renal Disease, n/N (%)	22/107 (20.6)	136/504 (27.0)	85/257 (33.1)
Chronic Liver Disease, n/N (%)	4/107 (3.7)	18/504 (3.6)	5/257 (1.9)
Solid Cancer, n/N (%)	20/107 (18.7)	111/504 (22.0)	48/257 (18.7)
Hematological Cancer, n/N (%)	6/107 (5.6)	18/504 (3.6)	10/257 (3.9)
Solid organ transplantation, n/N (%)	2/107 (1.9)	7/504 (1.4)	6/257 (2.3)
Chronic Immunosuppression, n/N (%)	8/107 (7.5)	18/504 (3.6)	14/257 (5.4)
Pregnancy, n/N (%)	5/107 (4.7)	0/504 (0.0)	1/257 (0.4)
Socio-economic status			
Education level, n/N (%)			
Low	41/72 (56.9)	300/445 (67.4)	155/235 (66.0)
Middle	24/72 (33.3)	87/445 (19.6)	58/235 (24.7)
High	7/72 (9.7)	58/445 (13.0)	22/235 (9.4)
Income category, n/N (%)			
Low income	55/94 (58.5)	269/479 (56.2)	146/251 (58.2)
Middle income	32/94 (34.0)	147/479 (30.7)	75/251 (29.9)
High income	7/94 (7.4)	63/479 (13.2)	30/251 (12.0)
Population density ^a^, median (IQR)	2591 (484–2591)	798 (390–2591)	941 (357–2591)
Median taxable income per capita ^b^, median (IQR)	23,807 (23,807–27,514)	27,075 (23,807–28,985)	26,892 (23,807–28,855)
Exposure			
Hospital-acquired infection, n/N (%)	7/106 (6.6)	33/498 (6.6)	15/256 (5.9)
Documented previous infections ^c^, n/N (%)	8/107 (7.5)	12/504 (2.4)	9/257 (3.5)
Hospital characteristics			
Mean ICU occupancy during hospital stay, median (IQR)	13 (7–20)	8 (4–14)	9 (5–16)
Clinical outcomes			
Severe ^d^ COVID-19, n/N (%)	29/106 (27.4)	98/501 (19.6)	40/256 (15.6)
ICU admission, n/N (%)	18/107 (16.8)	54/504 (10.7)	19/257 (7.4)
In-hospital mortality, n/N (%)	17/105 (16.2)	57/500 (11.4)	30/256 (11.7)
ARDS, n/N (%)	4/107 (3.7)	18/504 (3.6)	6/257 (2.3)
Hospital length of stay (days), median (IQR)	9 (4–14)	9 (5–16)	9 (5–17)

^a^ Population density at the patient’s place of residence (postal code level); ^b^ median net taxable income per capita at the patient’s place of residence (postal code level); ^c^ number of patients with one or multiple documented previous infections, i.e., with the date of diagnosis of the SARS-CoV-2 infection causing hospitalization more than 60 days after the date of sample collection from a previous documented positive COVID-19 test (RT-PCR or AG); ^d^ admission to an intensive care unit (ICU), occurrence of acute respiratory distress syndrome (ARDS), or in-hospital mortality.

**Table 2:** Counterfactual risk of potential outcomes under different vaccination schedules (R, in %), risk difference (RD) and relative risk (RR) estimates, and 95% confidence interval (CI) within a multi-center study estimating the effect of the SARS-CoV-2 vaccination schedule (primary vaccination plus mRNA booster vaccination versus primary vaccination without booster vaccination) on severe clinical outcomes (severe COVID-19, ICU admission, in-hospital mortality) of BA.1, BA.2 or BA.4/5 Omicron infection (identified through linkage with WGS-confirmed lineage data) among hospitalized COVID-19 patients in Belgium.

	Primary Vaccination, without Booster Vaccination (Control Group)	Primary Vaccination, Plus mRNA Booster Vaccination (Intervention Group)	Intervention Effect	
	R [95% CI]	R [95% CI]	RD [95% CI]	RR [95% CI]
Severe COVID-19	0.31 [0.19; 0.42]	0.18 [0.15; 0.21]	−0.13 [−0.24; −0.01]	0.59 [0.33; 0.85]
ICU admission	0.14 [0.06; 0.21]	0.10 [0.08; 0.12]	−0.04 [−0.11; 0.04]	0.74 [0.22; 1.26]
In-hospital mortality	0.24 [0.12; 0.35]	0.11 [0.09; 0.13]	−0.13 [−0.25; 0.00]	0.47 [0.15; 0.79]

**Table 3:** Counterfactual risk of potential outcomes under intervention (R, in %), risk difference (RD) and relative risk (RR) estimates, and 95% confidence interval (CI) predicted with the Omicron sub-lineage as observed (overall, identified through linkage with WGS test results) and under exposure to Omicron sub-lineage BA.1, BA.2 and BA.4/5, separately, within a multi-center cohort study estimating the effect of the SARS-CoV-2 vaccination schedule (heterologous versus homologous prime-boost vaccination) on severe clinical outcomes (severe COVID-19, ICU admission, in-hospital mortality; different outcomes indicated in bold) of BA.1, BA.2 or BA.4/5 Omicron infection (identified through linkage with WGS-confirmed lineage data) among hospitalized COVID-19 patients in Belgium.

**Outcome: Severe COVID-19**				
	Homologous Prime-Boost Scheme (Control Group)	Heterologous Prime-Boost Scheme (Intervention Group)	Intervention Effect	
	R [95% CI]	R [95% CI]	RD [95% CI]	RR [95% CI]
Overall	0.20 [0.16; 0.23]	0.16 [0.11; 0.20]	−0.04 [−0.10; 0.02]	0.80 [0.53; 1.07]
Omicron BA.1	0.19 [0.12; 0.27]	0.13 [0.06; 0.20]	−0.07 [−0.16; 0.03]	0.65 [0.22; 1.07]
Omicron BA.2	0.19 [0.13; 0.24]	0.14 [0.08; 0.21]	−0.04 [−0.13; 0.04]	0.77 [0.32; 1.23]
Omicron BA.4/5	0.21 [0.13; 0.29]	0.21 [0.10; 0.33]	0.00 [−0.12; 0.12]	1.01 [0.41; 1.61]
**Outcome: ICU admission**				
	Homologous prime-boost scheme (control group)	Heterologous prime-boost scheme (intervention group)	Intervention effect	
	R [95% CI]	R [95% CI]	RD [95% CI]	RR [95% CI]
Overall	0.11 [0.08; 0.13]	0.08 [0.04; 0.11]	−0.03 [−0.07; 0.02]	0.73 [0.34; 1.12]
Omicron BA.1	0.08 [0.03; 0.13]	0.04 [0.00; 0.08]	−0.04 [−0.10; 0.02]	0.51 [0.00; 1.15]
Omicron BA.2	0.10 [0.06; 0.15]	0.06 [0.01; 0.11]	−0.04 [−0.11; 0.02]	0.59 [0.00; 1.19]
Omicron BA.4/5	0.15 [0.08; 0.23]	0.17 [0.06; 0.28]	0.02 [−0.10; 0.13]	1.12 [0.27; 1.97]
**Outcome: In-hospital mortality**				
	Homologous prime-boost scheme (control group)	Heterologous prime-boost scheme (intervention group)	Intervention effect	
	R [95% CI]	R [95% CI]	RD [95% CI]	RR [95% CI]
Overall	0.12 [0.09; 0.15]	0.11 [0.07; 0.15]	0.00 [−0.05; 0.04]	0.96 [0.55; 1.38]
Omicron BA.1	0.14 [0.07; 0.20]	0.11 [0.04; 0.18]	−0.03 [−0.11; 0.06]	0.81 [0.18; 1.45]
Omicron BA.2	0.12 [0.07; 0.16]	0.11 [0.05; 0.16]	−0.01 [−0.09; 0.06]	0.89 [0.23; 1.56]
Omicron BA.4/5	0.09 [0.03; 0.15]	0.13 [0.04; 0.23]	0.04 [−0.06; 0.14]	1.42 [0.00; 2.53 × 10^5^]

## Data Availability

The individual level datasets generated or analyzed during the current study do not fulfill the requirements for open data access. The data is too dense and comprehensive to preserve patient privacy. The data of the individual data sources (Clinical Hospital Survey, Vaccinnet+, COVID-19 TestResult Database, StatBel, Surge Capacity Survey, and Common Base Registry for HealthCare Actor) within the LINK-VACC project are kept in the pseudonymized environment of healthdata.be and a link between the individual data in each of them takes place thanks to the use of a pseudonymized national reference number managed by healthdata.be under a “project mandate”. A “project mandate” consists of a group of individuals, a group of variables and a time period. Access rights to the pseudonymized data in the healthdata.be data warehouse are granted ad nominatum for the scientists involved in the surveillance activities at Sciensano. External investigators with a request for selected data should fill in the data request form (https://epistat.sciensano.be/datarequest/, accessed on 16 December 2022). Depending on the type of desired data (anonymous or pseudonymized), the provision of data will have to be assessed by the Belgian Information Security Committee Social Security & Health based on legal and ethical regulations, and is outlined in a data transfer agreement with the data owner (Sciensano).

## Supplementary Materials

The following supporting information can be downloaded at: https://www.mdpi.com/article/10.3390/vaccines11020378/s1; Supplementary A Table S1: Protocol of the target randomized control trials and target trial emulation by using observational data; Supplementary B: Directed Acyclic Graph specification Dagitty; Supplementary C Table S2: Counterfactual risk of potential outcomes under different vaccination schedules (R, in %), risk difference (RD) and relative risk (RR) estimates, and 95% confidence interval (CI) within a multi-center study estimating the effect of the SARS-CoV-2 vaccination schedule (primary vaccination plus mRNA booster vaccination versus primary vaccination without booster vaccination) on severe clinical outcomes (severe COVID-19, ICU admission, in-hospital mortality) of BA.1, BA.2 or BA.4/5 Omicron infection (identified by the time period of COVID-19 diagnosis) among hospitalized COVID-19 patients in Belgium; Supplementary D table S3: Differences between estimated risk differences (RDs) or relative risks (RRs) under exposure of different Omicron sub-lineages, and 95% confidence intervals (CI), for the different severe clinical outcomes (severe COVID-19, ICU admission, in-hospital mortality); Supplementary E table S4: Counterfactual risk of potential outcomes under intervention (R, in %), risk difference (RD) and relative risk (RR) estimates, and 95% confidence interval (CI) predicted with the Omicron sub-lineage as observed (overall, identified through linkage with WGS test results) and under exposure to Omicron sub-lineage BA.1, BA.2 and BA.4/5 separately, within a multi-center cohort study investigating the effect of the SARS-CoV-2 vaccination schedule (heterologous versus homologous prime-boost vaccination) on severe clinical outcomes (severe COVID-19, ICU admission, in-hospital mortality) of BA.1, BA.2 or BA.4/5 Omicron infection among hospitalized COVID-19 patients in Belgium; Supplementary F table S5: Differences between estimated risk differences (RDs) or relative risks (RRs) under exposure of different Omicron sub-lineages, and 95% confidence intervals (CI), for the different severe clinical outcomes (severe COVID-19, ICU admission, in-hospital mortality).
