# Supplementary material for: Homologous and Heterologous Prime-Boost Vaccination: Impact on Clinical Severity of SARS-CoV-2 Omicron Infection among Hospitalized COVID-19 Patients in Belgium
Source: Vaccines (Basel). 2023 Feb 7;11(2):378. doi: 10.3390/vaccines11020378 (PMC9961733; doi:10.3390/vaccines11020378)
Supplement: Supplementary file 1 [file vaccines-11-00378-s001.zip › vaccines-2177074-supplementary.pdf]

Supplementary

Supplement to:

Homologous and heterologous prime-boost vaccination: impact on clinical severity of SARS-CoV-2 Omicron infection among hospitalized COVID-19 patients in Belgium

Description:

This Supplementary provides readers with supplementary information about the analysis and manuscript.

Supplementary A: Target trial emulation protocol

Table S1. Protocol of the target randomized control trials and target trial emulation by using observational data

Protocol Component	Target Trial Specification	Target Trial Emulation
**Eligibility criteria**	• Individuals that acquired a SARS-CoV-2 infection (either community- or hospital-acquired, detected by an RT-PCR or AG laboratory test) more than 14 days after intervention (i.e., more than 14 days after receiving the last dose of their vaccination schedule) and COVID-19 disease progression of this infection requiring COVID-19 related hospital care<br>• **Enrolment period**: From the start of the Belgian booster vaccination campaign until the end of November 2022<br>• **Age**: adults (age >= 18)<br>• **SARS-CoV-2 variant of infection**: Patients infected with the Omicron variant (WGS-confirmed)	• Patients treated for COVID-19 in a Belgian hospital and with an admission form reported in the Clinical Hospital Survey (CHS) were considered. Patients without a laboratory-confirmed (RT-PCR or AG) SARS-CoV-2 infection and who were not tested because they experienced COVID-19 symptoms (i.e., in a screening context) were excluded. Patients with a missing pseudonymized national registry number were excluded. Transferred or readmitted patients, and patients admitted to a psychiatric hospital or a hospital without an intensive care unit (ICU) are excluded. Patients with an unknown date of diagnosis, starting date of COVID-19 related hospital care or date of discharge are not included, as well as patients diagnosed with COVID-19 more than 20 days before hospital admission or after hospital discharge.<br>• **Enrolment period**: Patients hospitalized for COVID-19 in a Belgian hospital between 1 September 2021 (i.e., the start of the Belgian booster vaccination campaign) and 23 November 2022 (i.e., the date of data extraction minus two weeks to allow for sufficient follow-up time). Patients admitted outside this enrolment period are excluded.<br>• **Age**: adults (age >= 18)<br>• **SARS-CoV-2 variant of infection**: Patients infected with the Omicron sub-lineages BA.1, BA.2, BA.4 or BA.5 as identified by linkage with WGS-confirmed lineage data from the COVID-19 TestResult database in the main analysis and defined by the time period of diagnosis (10 January 2022 – 27 February 2022, 26 March 2022 – 19 May 2022 and 17 June 2022 – 11 November 2022 for BA.1, BA.2 and BA.4/5 respectively) in the sensitivity analysis. Patients without a linked WGS-confirmed lineage or with a linked WGS-confirmed lineage different from Omicron BA.1,

		BA.2, BA.4 or BA.5 were excluded from the study population in the main analysis. Patients with a COVID-19 diagnosis date outside these predefined periods were excluded from the study population in the sensitivity analysis.
Intervention strategies	Target trial 1:<br>1) Intervention with mRNA (2 doses of BNT162b2 or mRNA-1273) or viral vector (1 dose of Ad26.COV2.S or 2 doses of ChAdOx1-S) primary vaccination plus an mRNA (BNT162b2 or mRNA-1273) booster vaccination<br>2) Intervention with mRNA (2 doses of BNT162b2 or mRNA-1273) or viral vector (1 dose of Ad26.COV2.S or 2 doses of ChAdOx1-S) primary vaccination (no booster vaccination)	Same as in the target trial.
	Target trial 2:<br>1) Intervention with a heterologous prime-boost vaccination, defined as a viral vector (1 dose of Ad26.COV2.S or 2 doses of ChAdOx1-S) primary vaccination and mRNA (BNT162b2 or mRNA-1273) booster vaccination<br>2) Intervention with a homologous prime-boost vaccination, defined as an mRNA (2 doses of BNT162b2 or mRNA-1273) primary vaccination and mRNA (BNT162b2 or mRNA-1273) booster vaccination	Same as in the target trial.
**Assignment procedures**	Participants are randomly assigned to an intervention arm. The participants are not assigned blindly, thus will be aware of the intervention strategy to which they have been assigned.	Vaccination status ascertainment based on linkage with the Vaccinnet+ registry. Vaccination status was ascertained on the date of the COVID-19 diagnosis. For a vaccination dose to be considered for vaccination status ascertainment, it has to be administered at least 14 days before the date of COVID-19 diagnosis.<br><br>Randomization is mimicked by conditioning on a set of factors that are assumed to confound the exposure-outcome pathway (as identified through construction of a Directed Acyclic Graph), resulting in comparable (exchangeable) intervention groups. We assume that individuals are exchangeable within levels of the following patient characteristics: patient's comorbidities, age, gender, pregnancy status, host

		genetics, socio-economic status (SES), previous infection(s), whether the patient is a nursing home resident, whether the patient is a healthcare worker, the hospital of admission and ICU load during COVID-19 related hospital stay, whether the patient had a hospital-acquired infection, treatment strategies used during the time of admission, and Omicron sub-lineage of infection. The adjustment is performed by adjusting for these covariates in the logarithmic regression model.
**Follow-up period**	• Follow-up starts at the start of the COVID-19 related hospital care<br>• Follow-up ends when the patient is discharged from the hospital	• Follow-up starts at hospitalization due to SARS-CoV-2 infection.<br>• Follow-up ends when the patient is discharged from the hospital
**Outcome**	Severe COVID-19, ICU transfer, in-hospital mortality	Severe COVID-19, ICU transfer, in-hospital mortality
**Causal contrast of interest**	Per-protocol effect	Observational analogue of the per-protocol effect

Supplementary B: Directed Acyclic Graph specification Dagitty

```
dag {
	bb="0,0,1,1"
	"Clinical severity" [outcome,pos="0.682,0.728"]
	"Healthcare worker" [adjusted,pos="0.514,0.291"]
	"Host genetics" [latent,pos="0.521,0.630"]
	"ICU load" [adjusted,pos="0.840,0.507"]
	"Nursing home resident" [adjusted,pos="0.516,0.200"]
	"Omicron sub-lineage" [adjusted,pos="0.540,0.805"]
	"Place of residence" [pos="0.513,0.036"]
	"Previous infection" [adjusted,pos="0.147,0.738"]
	"SARS-CoV-2 vaccination scheme" [exposure,pos="0.399,0.735"]
	"Treatment strategies" [latent,pos="0.529,0.886"]
	Age [adjusted,pos="0.518,0.434"]
	Comorbidities [adjusted,pos="0.520,0.543"]
	Gender [adjusted,pos="0.626,0.453"]
	HAI [adjusted,pos="0.667,0.800"]
	Hospital [adjusted,pos="0.840,0.614"]
	Pregnancy [adjusted,pos="0.518,0.368"]
	SES [adjusted,pos="0.514,0.124"]
	Time [pos="0.338,0.803"]
	"Healthcare worker" -> "Clinical severity"
	"Healthcare worker" -> "Previous infection" [pos="0.323,0.335"]
	"Healthcare worker" -> "SARS-CoV-2 vaccination scheme"
	"Host genetics" -> "Clinical severity"
	"Host genetics" -> "Previous infection"
	"Host genetics" -> Comorbidities
	"ICU load" -> "Clinical severity"
	"Nursing home resident" -> "Clinical severity"
	"Nursing home resident" -> "Previous infection" [pos="0.265,0.323"]
	"Nursing home resident" -> "SARS-CoV-2 vaccination scheme"
	"Omicron sub-lineage" -> "Clinical severity"
	"Place of residence" -> "ICU load"
	"Place of residence" -> "Previous infection" [pos="0.130,0.186"]
	"Place of residence" -> "SARS-CoV-2 vaccination scheme"
	"Place of residence" -> Hospital
	"Place of residence" <-> SES
	"Previous infection" -> "Clinical severity" [pos="0.438,0.990"]
	"Previous infection" -> "SARS-CoV-2 vaccination scheme"
	"SARS-CoV-2 vaccination scheme" -> "Clinical severity"
	"Treatment strategies" -> "Clinical severity"
	Age -> "Clinical severity"
	Age -> "Nursing home resident" [pos="0.334,0.367"]
	Age -> "Previous infection" [pos="0.321,0.421"]
	Age -> "SARS-CoV-2 vaccination scheme"
	Age -> Comorbidities
	Age -> Pregnancy
	Comorbidities -> "Clinical severity"
	Comorbidities -> "Previous infection" [pos="0.339,0.525"]
	Comorbidities -> "SARS-CoV-2 vaccination scheme"
	Gender -> "Clinical severity"
	Gender -> "Healthcare worker"
	Gender -> "Previous infection" [pos="0.322,0.487"]
	Gender -> Age
	Gender -> Comorbidities [pos="0.583,0.498"]
	Gender -> Pregnancy
	HAI -> "Omicron sub-lineage"
	HAI -> Comorbidities
	Hospital -> "Clinical severity"
	Hospital -> "ICU load"
```

```
    Pregnancy -> "Clinical severity"
    Pregnancy -> "Previous infection" [pos="0.333,0.379"]
    Pregnancy -> "SARS-CoV-2 vaccination scheme"
    SES -> "Clinical severity"
    SES -> "Previous infection" [pos="0.210,0.168"]
    SES -> "SARS-CoV-2 vaccination scheme"
    Time -> "ICU load"
    Time -> "Omicron sub-lineage"
    Time -> "Previous infection"
    Time -> "SARS-CoV-2 vaccination scheme"
    Time -> "Treatment strategies"
}
```

Supplementary C: Sensitivity analysis – Target Trial I: Primary vaccination plus mRNA booster vaccination *versus* primary vaccination without booster vaccination

Table S2. Counterfactual risk of potential outcomes under different vaccination schedules (R, in %), risk difference (RD) and relative risk (RR) estimates, and 95% confidence interval (CI) within a multi-center study estimating the effect of the SARS-CoV-2 vaccination schedule (primary vaccination plus mRNA booster vaccination *versus* primary vaccination without booster vaccination) on severe clinical outcomes (severe COVID-19, ICU admission, in-hospital mortality) of BA.1, BA.2 or BA.4/5 Omicron infection (identified by the time period of COVID-19 diagnosis) among hospitalized COVID-19 patients in Belgium.

	**Primary vaccination, without booster vaccination (control group)**	**Primary vaccination, plus mRNA booster vaccination (intervention group)**	**Intervention effect**	
	R [95% CI]	R [95% CI]	RD [95% CI]	RR [95% CI]
Severe COVID-19	0.20 [0.17; 0.23]	0.15 [0.14; 0.16]	-0.05 [-0.08; -0.02]	0.75 [0.61; 0.88]
ICU admission	0.08 [0.06; 0.10]	0.07 [0.06; 0.07]	-0.01 [-0.03; 0.01]	0.87 [0.63; 1.11]
In-hospital mortality	0.15 [0.12; 0.18]	0.10 [0.09; 0.11]	-0.05 [-0.08; -0.02]	0.67 [0.51; 0.82]

Supplementary D: Main analysis – Target Trial II: Primary vaccination plus mRNA booster vaccination *versus* primary vaccination without booster vaccination

Table S3. Differences between estimated risk differences (RDs) or relative risks (RRs) under exposure of different Omicron sub-lineages, and 95% confidence intervals (CI), for the different severe clinical outcomes (severe COVID-19, ICU admission, in-hospital mortality).

	Additive scale			Multiplicative scale		
	$RD_{BA.2}$ - $RD_{BA.1}$	$RD_{BA.4/5}$ - $RD_{BA.2}$	$RD_{BA.4/5}$ - $RD_{BA.1}$	$RR_{BA.2}$ - $RR_{BA.1}$	$RR_{BA.4/5}$ - $RR_{BA.2}$	$RR_{BA.4/5}$ - $RR_{BA.1}$
Severe COVID-19	0.03 [-0.10; 0.16]	0.04 [-0.11; 0.19]	0.07 [-0.08; 0.22]	0.13 [-0.49; 0.75]	0.24 [-0.53; 1.00]	0.37 [-0.37; 1.10]
ICU admission	0.00 [-0.09; 0.08]	0.06 [-0.07; 0.19]	0.06 [-0.07; 0.18]	0.09 [-0.79; 0.97]	0.52 [-0.52; 1.57]	0.61 [-0.44; 1.66]
In-hospital mortality	0.01 [-0.10; 0.13]	0.05 [-0.07; 0.18]	0.06 [-0.07; 0.19]	0.08 [-0.84; 1.00]	0.52 [-2.53e+05; 2.53e+05]	0.60 [-2.53e+05; 2.53e+05]

Supplementary E: Sensitivity analysis – Target Trial II: Heterologous *versus* homologous prime-boost vaccination

Table S4. Counterfactual risk of potential outcomes under intervention (R, in %), risk difference (RD) and relative risk (RR) estimates, and 95% confidence interval (CI) predicted with the Omicron sub-lineage as observed (overall, identified through linkage with WGS test results) and under exposure to Omicron sub-lineage BA.1, BA.2 and BA.4/5 separately, within a multi-center cohort study investigating the effect of the SARS-CoV-2 vaccination schedule (heterologous *versus* homologous prime-boost vaccination) on severe clinical outcomes (severe COVID-19, ICU admission, in-hospital mortality) of BA.1, BA.2 or BA.4/5 Omicron infection among hospitalized COVID- 19 patients in Belgium.

**Outcome: Severe COVID-19**				
	Homologous prime-boost scheme (control group)	Heterologous prime-boost scheme (intervention group)	Intervention regimen	
	R [95% CI]	R [95% CI]	RD [95% CI]	RR [95% CI]
Overall	0.15 [0.14; 0.16]	0.16 [0.14; 0.17]	0.01 [-0.01 ; 0.03]	1.06 [0.91; 1.21]
Omicron BA.1	0.16 [0.14; 0.18]	0.18 [0.14; 0.21]	0.02 [-0.02; 0.05]	1.10 [0.85; 1.35]
Omicron BA.2	0.14 [0.12; 0.16]	0.15 [0.12; 0.19]	0.01 [-0.03; 0.05]	1.09 [0.81; 1.36]
Omicron BA.4/5	0.14 [0.12; 0.16]	0.14 [0.11; 0.17]	0.00 [-0.04; 0.03]	0.98 [0.72; 1.24]
**Outcome: ICU admission**				
	Homologous prime-boost scheme (control group)	Heterologous prime-boost scheme (intervention group)	Intervention regimen	
	R [95% CI]	R [95% CI]	RD [95% CI]	RR [95% CI]
Overall	0.06 [0.06; 0.07]	0.06 [0.05; 0.08]	0.00 [-0.01; 0.02]	1.02 [0.78; 1.26]
Omicron BA.1	0.06 [0.04; 0.07]	0.06 [0.04; 0.09]	0.01 [-0.02; 0.03]	1.11 [0.69; 1.54]
Omicron BA.2	0.07 [0.05; 0.09]	0.06 [0.04; 0.08]	-0.01 [-0.04; 0.02]	0.88 [0.49; 1.27]
Omicron BA.4/5	0.07 [0.05; 0.08]	0.07 [0.04; 0.09]	0.00 [-0.02; 0.03]	1.05 [0.62; 1.47]
**Outcome: In-hospital mortality**				
	Homologous prime-boost scheme (control group)	Heterologous prime-boost scheme (intervention group)	Intervention regimen	
	R [95% CI]	R [95% CI]	RD [95% CI]	RR [95% CI]
Overall	0.10 [0.09; 0.11]	0.10 [0.09; 0.12]	0.00 [-0.02; 0.02]	1.01 [0.83; 1.19]
Omicron BA.1	0.12 [0.10; 0.14]	0.12 [0.10; 0.15]	0.01 [-0.03; 0.04]	1.06 [0.77; 1.35]
Omicron BA.2	0.09 [0.08; 0.11]	0.10 [0.07; 0.12]	0.00 [-0.03; 0.03]	1.02 [0.68; 1.35]
Omicron BA.4/5	0.10 [0.07; 0.12]	0.09 [0.06; 0.11]	0.00 [-0.04; 0.02]	0.93 [0.61; 1.25]

Supplementary F: Sensitivity analysis – Target Trial II: Heterologous *versus* homologous prime-boost vaccination

Table S5. Differences between estimated risk differences (RDs) or relative risks (RRs) under exposure of different Omicron sub-lineages, and 95% confidence intervals (CI), for the different severe clinical outcomes (severe COVID-19, ICU admission, in-hospital mortality).

	Additve scale			Multiplicative scale		
	$RD_{BA.2}$ - $RD_{BA.1}$	$RD_{BA.4/5}$ - $RD_{BA.2}$	$RD_{BA.4/5}$ - $RD_{BA.1}$	$RR_{BA.2}$ - $RR_{BA.1}$	$RR_{BA.4/5}$ - $RR_{BA.2}$	$RR_{BA.4/5}$ - $RR_{BA.1}$
Severe COVID-19	0.00 [-0.06; 0.05]	-0.01 [-0.07; 0.04]	-0.02 [-0.07; 0.03]	-0.02 [-0.38; 0.35]	-0.10 [-0.48; 0.28]	-0.12 [-0.47; 0.24]
ICU admission	-0.01 [-0.05; 0.02]	0.01 [-0.03; 0.05]	0.00 [-0.04; 0.03]	-0.23 [-0.80; 0.34]	0.17 [-0.41; 0.75]	-0.07 [-0.67; 0.54]
In-hospital mortality	0.00 [-0.05; 0.04]	-0.01 [-0.05; 0.04]	-0.01 [-0.06; 0.03]	-0.02 [-0.46; 0.41]	-0.08 [-0.55; 0.39]	-0.10 [-0.53; 0.33]
